# Assessment of the relationship between CT-severity scores, pulmonary artery diameters, and D-dimer/CRP ratios in COVID-19 patients

**DOI:** 10.3389/fmed.2026.1768527

**Published:** 2026-05-11

**Authors:** Ersin Doğanözü, Pınar Demir Gündoğmuş, Saadet İbiş, Turan Akdağ, Dilek Dülger, Emine Alp Meşe, Ayşe Ceren Doğanözü

**Affiliations:** 1 Department of Cardiology, Faculty of Medicine, Akdeniz University, Ankara, Türkiye; 2 Department of Sports Medicine, Gülhane Training and Research Hospital, Ankara, Türkiye; 3 Biochemistry Laboratory, Mayıs State Hospital, Ankara, Türkiye; 4 Meram Vocational School, Necmettin Erbakan University, Konya, Türkiye; 5 Department of Medical Microbiology, Bilkent City Hospital, Health Sciences University, Ankara, Türkiye; 6 Department of Infectious Diseases and Clinical Microbiology, Faculty of Medicine, Ankara Yıldırım Beyazıt University, Ankara, Türkiye; 7 Department of Anesthesiology and Reanimation, Ankara Etlik City Hospital, Ankara, Türkiye

**Keywords:** COVID-19, CT severity score, D-dimer/CRP ratio, pulmonary artery diameter, risk stratification

## Abstract

**Objectives:**

Coronavirus disease 2019 (COVID-19), caused by SARS-CoV-2, remains a global health concern. The computed tomography (CT) severity score is widely used as an objective measure of pulmonary involvement, while increased pulmonary artery (PA) diameter has emerged as a potential marker of disease severity. This study aimed to investigate the relationship between CT severity scores, PA diameter, and the D-dimer/CRP ratio, and to determine whether these parameters are independently associated with radiological severity.

**Materials and methods:**

This retrospective case-control study included 199 PCR-confirmed COVID-19 patients who underwent chest CT and laboratory evaluation. Sociodemographic and clinical data were obtained from hospital records. Laboratory parameters, including procalcitonin, C-reactive protein (CRP), D-dimer, and the D-dimer/CRP ratio, along with CT findings such as PA diameter, were analyzed. Patients were categorized according to CT severity score (0–25). Multivariable linear regression analysis was performed to identify independent predictors of CT severity.

**Results:**

Higher CT severity scores were significantly associated with increased levels of inflammatory and laboratory markers, including procalcitonin, CRP, D-dimer, and troponin (all *p* < 0.05). Procalcitonin showed a significant positive association with CT severity score. Significant differences between severity groups were also observed for age, hypertension, creatinine, hemoglobin, platelet count, D-dimer/CRP ratio, and PA diameter (all *p* < 0.05). A modest but significant positive correlation was found between PA diameter and CT severity score (*r* = 0.333, *p* < 0.001). In multivariable analysis, PA diameter (β = 0.194, *p* = 0.005), D-dimer/CRP ratio (β = −0.177, *p* = 0.007), age (β = 0.175, *p* = 0.043), and troponin (β = −0.280, *p* < 0.001) remained independently associated with CT severity score (R^2^ = 0.291).

**Conclusion:**

Higher CT severity scores are associated with increased PA diameter and lower D-dimer/CRP ratios. Integrating radiological parameters with inflammatory markers may improve early risk stratification in COVID-19 patients.

## Introduction

The coronavirus disease 2019 (COVID-19), caused by the SARS-CoV-2 virus, was first reported in Wuhan, Hubei, China, in 2019 and rapidly spread worldwide. Due to its high transmissibility, the disease evolved into a global health crisis and was declared a pandemic by the World Health Organization in March 2020 (1). In our country, from the first reported case on March 11, 2020, to May 12, 2022, a total of 14,147,500 cases and 38,940 deaths were reported (2). The rapid transmission and substantial mortality burden of COVID-19 have posed a significant threat to global public health (3).

Chest computed tomography (CT) has been widely used during emergency admissions because of its rapid availability and diagnostic utility. In addition to its role in diagnosis, CT is valuable for assessing disease severity (4). The most common radiological findings include ground-glass opacities, bilateral patchy consolidations, and predominantly peripheral involvement, particularly in the middle and lower lung zones (5). Furthermore, pulmonary artery (PA) diameter can be measured using chest CT and has recently attracted attention as a potential indicator of disease severity (6). Previous studies have suggested that an enlarged PA diameter may serve as a predictor of mortality in patients with COVID-19 pneumonia (7).

In parallel with radiological evaluation, several biochemical parameters have been investigated for their diagnostic and prognostic value. Procalcitonin, a precursor of calcitonin, has been reported to correlate positively with disease severity in COVID-19 (8). C-reactive protein (CRP), a well-established inflammatory marker, increases in response to systemic inflammation and tissue injury, including COVID-19 infection (9). D-dimer, a fibrin degradation product, is frequently elevated in COVID-19 patients and has been associated with thrombotic complications, particularly within the pulmonary microvasculature (10). Autopsy studies have demonstrated endothelial injury and microthrombosis in peripheral lung regions, even in the absence of large-vessel pulmonary embolism (7). The D-dimer/CRP ratio has recently been proposed as a novel prognostic index with potential predictive value for morbidity and mortality in various critical illnesses (11).

Although previous studies have independently evaluated CT severity scores, pulmonary artery diameter, and inflammatory markers in COVID-19, few investigations have simultaneously analyzed these parameters within a multivariable framework. In particular, the combined assessment of pulmonary artery diameter and the D-dimer/CRP ratio in relation to CT-based disease severity has not been adequately explored. By integrating structural radiological measurements with inflammatory indices, the present study aims to provide a more comprehensive evaluation of disease severity in patients with COVID-19. CT severity scoring is commonly used to quantify the extent of pulmonary involvement in COVID-19. In this system, each of the five lung lobes is visually scored for percentage involvement, yielding a total score ranging from 0 to 25. Mild disease is generally defined by limited ground-glass opacities with minimal lobar involvement (CT score < 7), moderate disease reflects more extensive bilateral involvement with increasing consolidation (CT score 7–18), and severe disease is characterized by diffuse lung involvement, dense consolidations, and possible architectural distortion (CT score > 18). These radiological patterns correlate with disease severity and are widely used for risk stratification.

## Materials and methods

### Patients

This retrospective case-control study examined patients diagnosed with COVID-19 (ICD-10 code U07.3) as of March 20, 2020, at Ankara 29 Mayıs State Hospital. Medical records of patients admitted with COVID-19 between March 31 and September 30, 2020, were reviewed retrospectively. Inclusion criteria encompassed patients over 18 years of age who tested positive for COVID-19 via PCR (Bio-speedy^®^ SARS CoV-2 Double Gene RT-qPCR, Bioeksen, Turkey) at the Ministry of Health’s reference laboratory. This molecular assay detects ORF1ab and N gene targets with a sensitivity limit of 200 genomes per mL. Sample extraction was performed using Vnat tubes, with RNase P serving as an internal control for normalization. Amplification was conducted on the Bio-Rad CFX96 system. A cycle threshold (Ct) value of less than 38 for RNase P, ORF1ab, and N genes was considered indicative of a positive result. Ct values for both viral targets were analyzed accordingly. Participants also underwent chest CT scans and provided requisite laboratory data during hospitalization. Patients exhibiting normal chest CT findings served as controls, while those with abnormal findings were categorized into two case groups based on chest severity scores. Data collected included sociodemographic variables such as age, gender, number and duration of hospitalizations, medication regimens, and laboratory parameters. Heart rates were obtained via electrocardiography, and chest CT scans were evaluated using the hospital’s electronic medical record system. Patients with incomplete data were excluded. Laboratory results, including procalcitonin, CRP, D-dimer, and the D-dimer/CRP ratio, along with peak levels of CRP and D-dimer during illness, were obtained from the hospital’s electronic records, utilizing files and imaging records of patients admitted to the COVID-19 outpatient clinic at Ankara 29 Mayıs State Hospital. Hospital admission was determined according to the national COVID-19 management guidelines in effect during the study period. Indications for hospitalization included the presence of respiratory symptoms (such as dyspnea), oxygen saturation below 94% on room air, radiological evidence of pneumonia, advanced age with comorbidities, or clinical deterioration requiring close monitoring. Patients without radiological involvement but with positive PCR results were hospitalized based on clinical judgment, comorbidity burden, and the need for observation during the early phase of the pandemic.

### Chest CT imaging protocol

A non-contrast-enhanced chest CT scan was performed using a 16-detector row scanner (BrightSpeed; GE Healthcare). All imaging procedures were carried out with the patient in the supine position during a single deep inspiratory breath hold. The imaging covered the region from the lung apex to the costophrenic angles. The specific acquisition parameters included: X-ray tube settings of 120 kVp and 350 mAs; rotation time of 0.5 s; pitch of 1.0; section thickness of 5 mm; and an interslice gap of 5 mm. Images were reconstructed utilizing a sharp convolution kernel with a slice thickness of 1.5 mm. Lung parenchyma evaluation employed window settings of 1000 to 2000 Hounsfield Units (HU) for the window width and −700 to −500 HU for the window level. All images were archived and analyzed through a Picture Archiving and Communication System (PACS).

### Chest CT image analysis

All patients underwent chest CT between days 4 and 7 after symptom onset. PA diameter measurements were performed by a single experienced radiologist who was blinded to laboratory data and clinical severity categories. Interobserver variability was not assessed due to the study’s retrospective design. The extent of pneumonic involvement observed on the chest CT during hospitalization was assessed by a radiologist, and disease severity was quantified using the CT severity score, which ranges from 0 to 25. Severity classification was as follows: mild (score < 7), moderate (score 7–18), and severe (score > 18), based on CT findings. The pulmonary artery (PA) diameter was measured at the proximal end of the bifurcation. The CT severity score was determined by counting the number of COVID-19-affected pulmonary segments and assigning a score to each segment. The total CT severity score ranged from 0 to 25 and was calculated by summing the individual segmental scores (5, 12, 13).

### Statistical analysis

The data analysis was conducted using IBM SPSS 25.0 (Armonk, NY: IBM Corp.) and MedCalc 20.218 (MedCalc Software bvba, Ostend, Belgium). To evaluate the study data, we utilized descriptive statistical methods such as frequency, percentage, mean, standard deviation, median, and min-max, as well as the Chi-Square test to compare qualitative data. We assessed the suitability of the data for normal distribution through the Kolmogorov-Smirnov test, skewness-kurtosis, and graphical methods like histogram, Q-Q Plot, and Boxplot.

For comparison between groups, we used the Independent Samples *t*-test and One-Way Anova test for normally distributed quantitative data, the Mann-Whitney U test, and the Kruskal-Wallis H test for non-normally distributed data. Furthermore, the relationships between variables were examined using the Pearson correlation test. A *p*-value of less than 0.05 was considered statistically significant.

A *post hoc* power analysis was performed based on the primary structural parameter of the study, namely the main pulmonary artery diameter, which served as the main radiological variable of interest. The analysis was conducted using G*Power software (version 3.1.9.7; Heinrich-Heine-Universität Düsseldorf, Germany) for a one-way ANOVA design with three groups (control, mild, and moderate/severe). The effect size (*f* = 0.34) was calculated from the observed differences in pulmonary artery diameter between groups (23.0 ± 2.7 mm, 24.1 ± 3.1 mm, and 25.9 ± 3.4 mm) and the pooled standard deviation (SD = 3.3). With a total sample size of 199 patients (n1 = 44, n2 = 84, n3 = 71) and α = 0.05, the achieved power was 99%.

The D-dimer/CRP ratio was analyzed as a secondary laboratory parameter.

## Results

During the study period, 520 patients were admitted with COVID-19, of whom 199 with available data were included in the analysis. The clinical characteristics of these patients are summarized in Table 1. The cohort comprised 100 females (49.7%), with comorbidities including hypertension (HT) in 64 patients, diabetes mellitus (DM) in 42 patients, coronary heart disease (CHD) in 10 patients, and chronic lung disease (CLD) in 16 patients. Among patients in the moderate–severe group (*n* = 71), 59 (83%) patients were classified as moderate and 12 (17%) as severe based on CT severity scores. *Post hoc* multiple-comparison tests were conducted to identify statistically significant differences between groups, with the detailed results presented in Table 1. These comparisons revealed statistically significant differences between groups in age, presence of comorbidities, hypertension, CRP, creatinine, hemoglobin, platelet count, troponin, procalcitonin, D-dimer levels, D-dimer/CRP ratio, and PA diameter (all *p* < 0.05) (Table 2). The control group had a lower prevalence of comorbidities than the other groups.

**TABLE 1:** Clinical characteristics of the participants.

Variable	Value
Sex, n (%)	
**Female**	100 (50.3)
**Male**	99 (49.7)
**Age (years)**	54.2 ± 17.1
Comorbidities, n (%)	
**No**	107 (53.8)
**Yes**	92 (46.2)
**Chronic lung disease (CLD)**	16 (8.0)
**Chronic heart disease (CHD)**	10 (5.0)
**Hypertension (HTN)**	64 (32.2)
**Diabetes mellitus**	42 (21.1)
**Cerebrovascular event (CVE)**	3 (1.5)
**Chronic kidney disease (CKD)**	3 (1.5)
**Chronic obstructive pulmonary disease (COPD)**	6 (3.0)
**Hypothyroidism**	13 (6.5)
**Hyperthyroidism**	0 (0.0)
Laboratory parameters	
**ALT (U/L)**	32.0 ± 29.5
**AST (U/L)**	32.9 ± 23.6
**CRP (mg/L)**	39.8 ± 38.6
**Creatinine (mg/dL)**	0.9 ± 0.3
**White blood cell count (×10^9^/L)**	5954.6 ± 2488.5
**Hemoglobin (g/dL)**	13.7 ± 1.7
**Platelet count (×10^3^/μL)**	214697.0 ± 71068.1
**Troponin (ng/mL)**	1.4 ± 2.7
**Procalcitonin (ng/mL)**	0.2 ± 0.6
**D-dimer (ng/mL)**	917.1 ± 900.5
**D-dimer/CRP ratio**	112.0 ± 295.5
**Heart rate on baseline ECG (bpm)**	80.6 ± 14.3
**Pulmonary artery diameter (mm)**	24.5 ± 3.3
**CT severity score**	6.5 ± 5.6
**Control group**	44 (22.1)
**Mild**	84 (42.2)
**Moderate +** severe	71 (35.7)
Mortality, n (%)	
**No**	193 (97.0)
**Yes**	6 (3.0)

*n*/%; mean ± SD/median (min-max); Add Dis, addition disease; CLD, chronic lung disease; CHD, chronic heart disease; HTN, hypertension; CVE, cerebrovascular event; CKD, chronic kidney disease; COPD, chronic obstructive pulmonary disease; ALT, alanine transaminase; AST, aspartate transaminase; CRP, C reactive protein; WBC, white blood cell.

**TABLE 2:** Comparisons between CT severity groups.

Variable	Control (*n* = 44)	Mild (*n* = 84)	Moderate–severe (*n* = 71)	*P*-value	*Post hoc* comparison
Sex, *n* (%)				0.784^a^	–
Female	21 (47.7)	41 (48.8)	38 (53.5)		
Male	23 (52.3)	43 (51.2)	33 (46.5)		
Age (years), mean ± SD	41.7 ± 18.0	54.0 ± 15.4	62.1 ± 13.8	<0.001^b^	1–2–3
Any comorbidity, *n* (%)	8 (18.2)	40 (47.6)	44 (62.0)	<0.001^a^	1–2–3
Hypertension, *n* (%)	5 (11.4)	30 (35.7)	29 (40.8)	0.003^a^	1–2–3
Diabetes mellitus, *n* (%)	5 (11.4)	17 (20.2)	20 (28.2)	0.097^a^	–
Chronic lung disease, *n* (%)	2 (4.5)	6 (7.1)	8 (11.3)	0.403^a^	–
Chronic heart disease, *n* (%)	2 (4.5)	3 (3.6)	5 (7.0)	0.607^a^	–
COPD, *n* (%)	1 (2.3)	3 (3.6)	2 (2.8)	0.913^a^	–
CKD, *n* (%)	0 (0.0)	1 (1.2)	2 (2.8)	0.461^a^	–
ALT (U/L), median (min–max)	18.0 (7.0–124.0)	24.0 (8.0–260.0)	27.0 (7.0–178.0)	0.054^c^	–
AST (U/L), median (min–max)	22.0 (10.0–75.0)	27.0 (12.0–188.0)	32.0 (15.0–225.0)	<0.001^c^	1 vs. 2–3
CRP (mg/L), mean ± SD	9.4 ± 8.1	36.5 ± 32.4	74.8 ± 49.9	<0.001^b^	1–2–3
Creatinine (mg/dL), median (min–max)	0.8 (0.4–1.1)	0.8 (0.4–2.0)	0.9 (0.5–3.0)	0.011^c^	1 vs. 2–3
WBC (×10^9^/L), mean ± SD	5.33 ± 1.75	5.34 ± 2.06	7.06 ± 2.93	<0.001^b^	3 vs. 1–2
Hemoglobin (g/dL), mean ± SD	14.2 ± 2.1	13.8 ± 1.5	13.2 ± 1.5	0.007^b^	1 vs. 3
Platelet (×10^3^/μL), mean ± SD	204.7 ± 54.4	199.2 ± 60.1	239.0 ± 84.9	0.001^b^	3 vs. 1–2
Troponin (ng/mL), median (min–max)	2.5 (0.0–13.6)	0.0 (0.0–25.3)	0.0 (0.0–9.7)	<0.001^c^	1–2–3
Procalcitonin (ng/mL), median (min–max)	0.0 (0.0–0.5)	0.0 (0.0–1.8)	0.1 (0.0–6.1)	<0.001^c^	3 vs. 1–2
D-dimer (ng/mL), median (min–max)	270.0 (150.0–5330.0)	370.0 (40.0–12600.0)	965.0 (170.0–8240.0)	<0.001^c^	3 vs. 1–2
D-dimer/CRP ratio, median (min–max)	63.3 (7.0–2375.0)	36.2 (2.1–2693.2)	17.2 (2.9–485.7)	<0.001^c^	1–2–3
Heart rate (bpm), mean ± SD	77.6 ± 12.5	80.8 ± 13.9	82.2 ± 15.6	0.281^b^	–
Pulmonary artery diameter (mm), mean ± SD	23.0 ± 2.7	24.1 ± 3.1	25.9 ± 3.4	<0.001^b^	3 vs. 1–2
Mortality, *n* (%)	0 (0.0)	2 (2.4)	4 (5.6)	0.207^a^	–

a, Chi-Square Test (*n*/%); b, One-Way Anova Test (Mean ± SD); c, Kruskal-Wallis H Test [Median (Min-Max)]; Add Dis, addition disease; CLD, chronic lung disease; CHD, chronic heart disease; HT, hypertension; CVE, cerebrovascular event; CKD, chronic kidney disease; COPD, chronic obstructive pulmonary disease; ALT, alanine transaminase; AST, aspartate transaminase; CRP, C reactive protein; WBC, white blood cell.

A modest but statistically significant positive correlation was observed between PA diameter and CT severity score (*r* = 0.333, *p* < 0.001).

**FIGURE 1:**
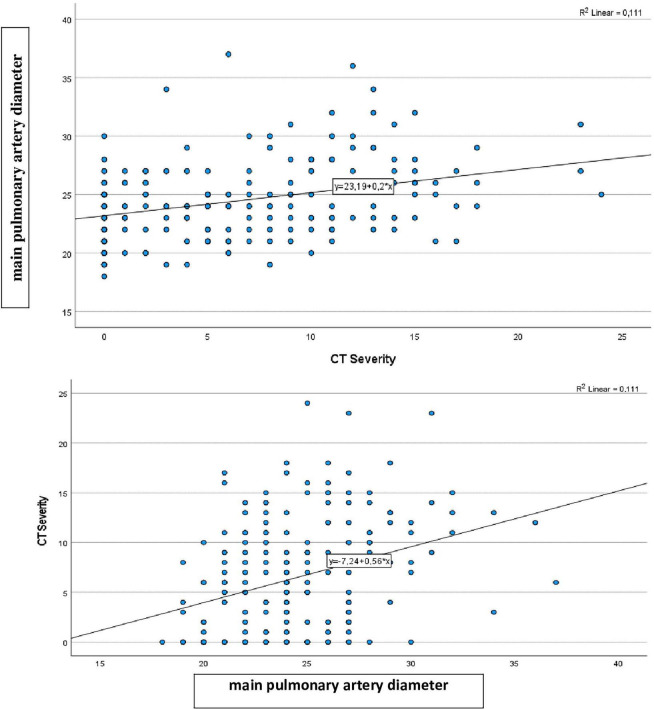
Correlation between main pulmonary artery diameter and CT severity. Two scatter plots each display a linear regression line. The first plot shows main pulmonary artery diameter versus CT severity, with a weak positive trend and equation y equals 23.19 plus 0.2x, R squared equals 0.111. The second plot shows CT severity versus main pulmonary artery diameter, with a similar trend, equation y equals 7.24 plus 0.56x, R squared equals 0.111. Blue dots represent individual data points.

Univariate correlation analyses were performed to assess the relationship between CT severity score and clinical, laboratory, and radiological variables. CT severity score showed significant positive correlations with age, CRP, D-dimer, procalcitonin, white blood cell count, platelet count, creatinine, and pulmonary artery diameter, while significant negative correlations were observed with hemoglobin, troponin, and the D-dimer/CRP ratio (all *p* < 0.05). Sex was not significantly associated with CT severity score in either univariate or multivariable analyses (*p* > 0.05). Detailed results are presented in Table 3, and the correlation between main pulmonary artery diameter and CT severity score is illustrated in Figure 1. Procalcitonin levels were also significantly associated with CT severity score. In univariate analysis, procalcitonin demonstrated a positive correlation with CT severity score (*r* = 0.246, *p* < 0.001), indicating that higher procalcitonin levels were observed in patients with more extensive pulmonary involvement. Additionally, procalcitonin levels differed significantly across CT severity groups, with higher values in the moderate-to-severe group compared to patients with mild or no radiological involvement. These findings suggest that procalcitonin may reflect the extent of inflammatory burden and disease severity in COVID-19. Procalcitonin was also independently associated with CT severity score in the multivariable model (β = 0.150, *p* = 0.020).

**TABLE 3:** Univariate correlations between studied variables and CT severity score.

Variable	R*	*P*-value
Age	0.413	<0.001
ALT	0.085	0.232
AST	0.260	<0.001
CRP	0.603	<0.001
Creatinine	0.258	<0.001
White blood cell count	0.399	<0.001
Hemoglobin	−0.226	0.001
Platelet	0.278	<0.001
Troponin	−0.342	<0.001
Procalcitonin	0.246	<0.001
D-dimer	0.340	<0.001
D-dimer/CRP ratio	−0.221	0.002
Heart rate	0.198	0.007
Pulmonary artery diameter	0.333	<0.001

*Pearson correlation test. ALT, alanine transaminase; AST, aspartate transaminase; CRP, C reactive protein; Computed Tomography.

Multivariable linear regression analysis was performed to determine independent predictors of CT severity score (Table 4). After adjustment for age, sex, comorbidities, pulmonary artery diameter, D-dimer/CRP ratio, and troponin levels, pulmonary artery diameter (β = 0.194, *p* = 0.005), D-dimer/CRP ratio (β = −0.177, *p* = 0.007), age (β = 0.175, *p* = 0.043), and troponin (β = −0.280, *p* < 0.001) remained independently associated with CT severity score. The overall regression model was statistically significant (R^2^ = 0.291, *F* = 12.590, *p* < 0.001) (Table 4).

**TABLE 4:** Multivariable linear regression analysis for predictors of CT severity score.

Predictor	Estimate	SE	Lower CI	Upper CI	t	*p*	Standardized β
Intercept^a^	−2.688	2.858	−8.329	2.953	−0.940	0.348	–
Sex	0.002	0.743	−1.464	1.468	0.003	0.998	0.000
Age	0.055	0.030	−0.003	0.114	1.872	0.063	0.167
Pulmonary artery diameter	0.274	0.119	0.039	0.508	2.306	0.022	0.159
Comorbidity	1.003	0.921	−0.815	2.820	1.089	0.278	0.178
D-dimer/CRP ratio	−0.003	0.001	−0.006	−0.001	−2.618	0.010	−0.170
Troponin	−0.574	0.135	−0.841	−0.307	−4.243	<0.001	−0.280
Procalcitonin	1.394	0.593	0.223	2.565	2.349	0.020	0.150

Model statistics: *R* = 0.555, R^2^ = 0.308, *F*(7,175) = 11.150, *p* < 0.001. ^a^Reference category. Sex was coded as 0 = female and 1 = male. Comorbidity was coded as 0 = no and 1 = yes.

## Discussion

This study found that the D-dimer-to-CRP ratio was significantly lower in patients with moderate-to-severe lung infection than in those with mild lung infection or individuals without pneumonia. Additionally, our findings indicate that the pulmonary artery diameter was significantly enlarged in patients with moderate to severe pulmonary infection relative to the control and mild groups. To our knowledge, limited data are available regarding the D-dimer/CRP ratio in COVID-19.

D-dimer serves as an escalating biomarker indicative of disrupted coagulation-thrombosis balance. Its levels begin to rise at the initial stages of COVID-19, with elevated D-dimer levels correlating with increased mortality rates (14). Typically, D-dimer elevation is observed in severe infections characterized by endothelial damage and coagulopathy. Multiple factors influence this process, including heightened cytokine production, increased leukocyte adhesion resulting from an exaggerated immune response, direct viral cytopathic effects, reduced nitric oxide availability, impaired endothelial repair mechanisms, and hypercoagulability (15). The activated coagulation system functions as a protective response, limiting infection dissemination; however, excessive activation may precipitate thrombosis or hemorrhage (16). D-dimer possesses high negative predictive value, making it a useful marker to exclude embolic events. Nonetheless, its positive predictive value in the context of severe COVID-19 remains uncertain, as levels may be elevated in the absence of thromboembolic phenomena (17). Elevated D-dimer levels observed in COVID-19 pneumonia have contributed to diagnostic ambiguity among clinicians. Differentiating pulmonary thromboembolism remains a critical objective in patients presenting with elevated D-dimer levels and hypoxemia. In this study, it was observed that inflammatory markers exhibited more significant elevation than D-dimer levels. Moreover, the D-dimer/CRP ratio was lower in patients with severe pneumonia compared to both the control group and those with mild disease. We hypothesize that the D-dimer/CRP ratio could serve as a valuable tool for differential diagnosis in emergency and intensive care settings.

Based on our study, we found a significant association between PA diameter and inflammatory markers such as CRP, white cell blood count, and procalcitonin, as well as with CT severity scores. This suggests a modest association between PA diameter and disease severity. The PA, being impacted by lung involvement, has been shown to undergo expansion during COVID-19. As the disease severity increases, there is a corresponding increase in inflammatory parameters. This may be attributed to activated immune pathways, potentially leading to reduced pulmonary capacity and elevated PA pressure (18). The development of increased pulmonary pressure in individuals with pneumonia may be attributed not only to the infection itself but also to undetected subsegmental microembolic foci (19). Recently, PA dilatation has also been found in non-coronavirus cases of pneumonia (6). In our investigation, we observed PA dilatation in the course of COVID-19 alongside a noteworthy correlation between PA diameter and increasing CT severity score. Unlike typical pneumonias, COVID-19 pneumonia initiates early and progresses rapidly, with the rate of pneumonic infiltration being indicative of disease severity. Prolonged hypoxia and extensive lung infiltration during COVID-19 progression are anticipated to result in elevated pulmonary vascular resistance (19). Notably, our study revealed that the moderate/severe group, characterized by a high CT severity score, exhibited larger PA dimensions, thus substantiating this hypothesis. Although the observed correlation between pulmonary artery diameter and CT severity score was modest in magnitude, it remained statistically significant after multivariable adjustment. This suggests that pulmonary artery enlargement may represent one component of disease severity rather than a standalone determinant. In addition to these findings, procalcitonin demonstrated a significant positive association with CT severity score in our study. Patients with higher CT severity scores exhibited elevated procalcitonin levels, suggesting a link between increased inflammatory burden and the extent of pulmonary involvement. Procalcitonin has been widely reported as a marker of disease severity in COVID-19, with higher levels associated with severe disease, secondary bacterial infection, and worse clinical outcomes (8). Previous studies, including recent evidence, have shown that elevated procalcitonin levels correlate with increased inflammatory response and may reflect a more advanced stage of disease (8, 20). Our findings are consistent with this evidence and further support the role of procalcitonin as a complementary biomarker in assessing radiological severity in COVID-19. The observed association between procalcitonin and CT severity score suggests that integrating biochemical markers with imaging findings may provide a more comprehensive evaluation of disease progression.

Since the onset of the COVID-19 pandemic, clinicians have generally asserted an increase in thromboembolic events among affected patients. It has been hypothesized that pulmonary artery dilation and elevated D-dimer levels observed during COVID-19 are attributable to pulmonary thromboembolism (PTE) (21). However, recent investigations indicate that the incidence of venous thromboembolism (VTE) in COVID-19 patients is comparable to that observed in other infectious diseases, approximately 7% (17). Conversely, some data suggest a VTE rate of approximately 50% in this patient population (22). The prevalence of VTE and PTE in COVID-19 remains a subject of ongoing debate. Clinicians often administer anticoagulation therapy to hospitalized patients and conduct monitoring for thromboembolic complications. Various studies have reported differing detection rates of VTE in the context of COVID-19 (17). A recent study within this setting found the rates of PTE and deep vein thrombosis (DVT) to be 16.5% and 14.8%, respectively. Notably, the study revealed that over half of the patients with PTE did not exhibit clinical signs of DVT, underscoring that COVID-19 predominantly affects the respiratory system (19). Another investigation reported a DVT rate of approximately 2%, with PTE rates reaching up to 16% (22). Given COVID-19’s primary impact on the respiratory system, reliance solely on DVT screening for PTE detection is inadvisable. Consequently, the use of objective diagnostic parameters is essential for accurate PTE assessment.

Numerous studies have examined the D-dimer cut-off for diagnosing pulmonary thromboembolism (PTE) in the context of COVID-19 (21, 23). Variations in patient populations, including comorbidities and disease severity, as well as differences in methodological approaches, have contributed to inconsistent findings. Some studies measured D-dimer levels at hospitalization, others at emergency room admission, and others focused on peak values during hospitalization. Moreover, the definitive D-dimer level indicative of PTE remains unidentified (24).

Patients may require hospitalization at any stage of the disease course. It is crucial to acknowledge that D-dimer levels assessed at the time of admission to emergency or outpatient settings may not consistently provide reliable information, regardless of disease severity. CRP serves as an important marker of disease severity in COVID-19, similarly to its role in other infections (18). Our findings suggest that the D-dimer/CRP ratio may provide complementary information regarding disease severity. The study revealed a significant correlation between CRP and D-dimer levels and the CT severity score, with CRP levels exhibiting a more marked increase. Therefore, the D-dimer/CRP ratio may represent a potentially useful adjunctive parameter when interpreted alongside radiological severity findings.

From a practical clinical standpoint, the integration of pulmonary artery diameter and the D-dimer/CRP ratio with CT severity scoring may provide a rapid and accessible method for early risk stratification. These parameters are routinely available at the time of hospital admission and may assist clinicians in identifying patients at increased risk of severe pulmonary involvement without the need for advanced imaging modalities such as CT pulmonary angiography. This approach may be particularly valuable in resource-limited settings or during periods of high patient volume.

Interestingly, troponin levels demonstrated an inverse association with CT severity score in the adjusted model. This finding may reflect the multifactorial nature of myocardial injury in COVID-19, which may occur independently of the extent of radiological pulmonary involvement. Myocardial injury in COVID-19 has been associated with systemic inflammation, microvascular dysfunction, and pre-existing cardiovascular conditions, and therefore may not directly parallel the degree of parenchymal lung involvement (25, 26). Further studies are warranted to clarify this relationship. Importantly, the associations between pulmonary artery diameter, D-dimer/CRP ratio, and CT severity score remained significant after multivariable adjustment, supporting an independent relationship beyond the influence of age and comorbid conditions. Due to the low mortality rate in our cohort (3%), outcome-based regression analyses were not feasible.

The following limitations should be acknowledged. First, the sample size was limited, and data were obtained through retrospective analysis. It is important to note that vascular embolism diagnoses in our patient cohort were not explicitly documented; therefore, the association between embolism and D-dimer elevation was only indirectly examined. Given that age differed significantly between groups, residual confounding cannot be excluded. Additionally, the small number of patients with high CT severity scores may have influenced the findings. Although the power analysis was based on pulmonary artery diameter as the primary radiological parameter, the significant associations observed for the D-dimer/CRP ratio in multivariable analysis further support the robustness of the findings. Importantly, the present findings should be interpreted within the context of observational data and do not imply causality. Additionally, chest CT scans were performed without intravenous contrast enhancement, which may have limited the accurate assessment of vascular structures and potential thromboembolic findings.

From a clinical perspective, these findings suggest that integrating pulmonary artery diameter and the D-dimer/CRP ratio with CT severity scoring may improve risk stratification at hospital admission. This combined radiological-biochemical approach may be particularly valuable in settings where access to advanced imaging modalities, such as CT pulmonary angiography, is limited or when clinical decision-making must rely on readily available parameters.

## Conclusion

Elevated D-dimer levels are a well-documented feature of COVID-19 and are frequently associated with thrombotic risk. However, definitive D-dimer cut-off values for diagnosing vascular thrombosis or embolism remain uncertain, as elevated levels may reflect systemic inflammation and disease progression rather than overt thromboembolism. In this context, our findings suggest that integrating pulmonary artery diameter and the D-dimer/CRP ratio with CT severity scoring may provide a more comprehensive assessment of disease burden. This combined radiological–biochemical approach could enhance risk stratification at hospital admission and support clinical decision-making using readily available parameters.

## Data Availability

The raw data supporting the conclusions of this article will be made available by the authors, without undue reservation.

## References

[1] Chauhan S. Comprehensive review of coronavirus disease 2019 (COVID-19). *Biomed J*. (2020) 43:334–40. 10.1016/j.bj.2020.05.023 32788071 PMC7263230

[2] Budak F Korkmaz Ş. COVID-19 pandemi sürecine yönelik genel bir değerlendirme: Türkiye örneği. *Sosyal Araştırmalar ve Yönetim Dergisi.* (2020) 1:62–79. 10.35375/sayod.738657

[3] Hippisley-Cox J Coupland CA Mehta N Keogh RH Diaz-Ordaz K Khunti K et al. Risk prediction of covid-19 related death and hospital admission in adults after covid-19 vaccination: national prospective cohort study. *BMJ*. (2021) 374:n2244. 10.1136/bmj.n2244 34535466 PMC8446717

[4] Yang R Li X Liu H Zhen Y Zhang X Xiong Q et al. Chest CT Severity Score: an Imaging Tool for Assessing Severe COVID-19. *Radiol Cardiothorac Imaging*. (2020) 2:e200047. 10.1148/ryct.2020200047 33778560 PMC7233443

[5] Hosseiny M Kooraki S Gholamrezanezhad A Reddy S Myers L. Radiology perspective of coronavirus disease 2019 (COVID-19): lessons from severe acute respiratory syndrome and middle east respiratory syndrome. *AJR Am J Roentgenol*. (2020) 214:1078–82. 10.2214/AJR.20.22969 32108495

[6] Lozano-Carrillo LC Alvarez-Lozada LA Fernández-Reyes BA Rodríguez-Alanís KV Montemayor-Martinez A de-la-Garza-Castro O et al. Main pulmonary artery diameter related to pneumonia severity. *Clin Anat*. (2024) 37:784–90. 10.1002/ca.24203 38984460

[7] Tastemur M Olcucuoğlu E Arik G Ates I Silay K. Pulmonary artery diameter and NT-proBNP in patients with Covid-19: predicting prognosis and mortality. *Afr Health Sci*. (2023) 23:553–64. 10.4314/ahs.v23i2.64 PMC10782310 38223639

[8] Hu R Han C Pei S Yin M Chen X. Procalcitonin levels in COVID-19 patients. *Int J Antimicrob Agents*. (2020) 56:106051. 10.1016/j.ijantimicag.2020.106051 32534186 PMC7286278

[9] Luan YY Yin CH Yao YM. Update Advances on C-Reactive Protein in COVID-19 and Other Viral Infections. *Front Immunol*. (2021) 12:720363. 10.3389/fimmu.2021.720363 34447386 PMC8382792

[10] Adam SS Key NS Greenberg CS. D-dimer antigen: current concepts and future prospects. *Blood*. (2009) 113:2878–87. 10.1182/blood-2008-06-165845 19008457

[11] Ullah W Thalambedu N Haq S Saeed R Khanal S Tariq S et al. Predictability of CRP and D-Dimer levels for in-hospital outcomes and mortality of COVID-19. *J Community Hosp Intern Med Perspect*. (2020) 10:402–8. 10.1080/20009666.2020.1798141 33235672 PMC7671719

[12] Pan F Ye T Sun P Gui S Liang B Li L et al. Time course of lung changes at chest CT during recovery from coronavirus disease 2019 (COVID-19). *Radiology*. (2020) 295:715–21. 10.1148/radiol.2020200370 32053470 PMC7233367

[13] Francone M Iafrate F Masci GM Coco S Cilia F Manganaro L et al. Chest CT score in COVID-19 patients: correlation with disease severity and short-term prognosis. *Eur Radiol*. (2020) 30:6808–17. 10.1007/s00330-020-07033-y 32623505 PMC7334627

[14] Shah S Shah K Patel SB Patel FS Osman M Velagapudi P et al. Elevated D-dimer levels are associated with increased risk of mortality in coronavirus disease 2019: a systematic review and meta-analysis. *Cardiol Rev*. (2020) 28:295–302. 10.1097/CRD.0000000000000330 33017364 PMC7437424

[15] Lin L Lu L Cao W Li T. Hypothesis for potential pathogenesis of SARS-CoV-2 infection-a review of immune changes in patients with viral pneumonia. *Emerg Microbes Infect*. (2020) 9:727–32. 10.1080/22221751.2020.1746199 32196410 PMC7170333

[16] Antoniak S. The coagulation system in host defense. *Res Pract Thromb Haemost*. (2018) 2:549–57. 10.1002/rth2.12109 30046760 PMC6046589

[17] Vandenbriele C Gorog DA. Screening for venous thromboembolism in patients with COVID-19. *J Thromb Thrombolysis*. (2021) 52:985–91. 10.1007/s11239-021-02474-8 34019231 PMC8137803

[18] Ponti G Maccaferri M Ruini C Tomasi A Ozben T. Biomarkers associated with COVID-19 disease progression. *Crit Rev Clin Lab Sci*. (2020) 57:389–99. 10.1080/10408363.2020.1770685 32503382 PMC7284147

[19] Yildiz M Yadigar S Yildiz BŞ Aladag NB Keskin O Ozer RS et al. Evaluation of the relationship between COVID-19 pneumonia severity and pulmonary artery diameter measurement. *Herz.* (2021) 46:56–62. 10.1007/s00059-020-05014-x 33433652 PMC7802061

[20] Zobel CM Wenzel W Krüger JP Baumgarten U Wagelöhner T Neumann N et al. Serum interleukin-6, procalcitonin, and C-reactive protein at hospital admission can identify patients at low risk for severe COVID-19 progression. *Front Microbiol*. (2023) 14:1256210. 10.3389/fmicb.2023.1256210 37937220 PMC10626435

[21] Suh YJ Hong H Ohana M Bompard F Revel MP Valle C et al. Pulmonary embolism and deep vein thrombosis in COVID-19: a systematic review and meta-analysis. *Radiology*. (2021) 298:E70–80. 10.1148/radiol.2020203557 33320063 PMC7745997

[22] Mohamed MFH Al-Shokri SD Shunnar KM Mohamed SF Najim MS Ibrahim SI et al. Prevalence of venous thromboembolism in critically Ill COVID-19 patients: systematic review and meta-analysis. *Front Cardiovasc Med*. (2021) 7:598846. 10.3389/fcvm.2020.598846 33585578 PMC7874113

[23] Kumarasamy S Kaman L Tandup C Thakur UK Savlania A. Periampullary carcinoma in a 13-year-old with microsatellite instability treated successfully with pancreaticoduodenectomy. *Cureus*. (2022) 14:e22139. 10.7759/cureus.22139 35308766 PMC8920813

[24] Ventura-Díaz S Quintana-Pérez JV Gil-Boronat A Herrero-Huertas M Gorospe-Sarasúa L Montilla J et al. A higher D-dimer threshold for predicting pulmonary embolism in patients with COVID-19: a retrospective study. *Emerg Radiol*. (2020) 27:679–89. 10.1007/s10140-020-01859-1 33025219 PMC7538266

[25] Guo T Fan Y Chen M Wu X Zhang L He T et al. Cardiovascular implications of fatal outcomes of patients with coronavirus disease 2019 (COVID-19). *JAMA Cardiol*. (2020) 5:811–8. 10.1001/jamacardio.2020.1017 32219356 PMC7101506

[26] Siripanthong B Nazarian S Muser D Deo R Santangeli P Khanji MY et al. Recognizing COVID-19-related myocarditis: the possible pathophysiology and proposed guideline for diagnosis and management. *Heart Rhythm*. (2020) 17:1463–71. 10.1016/j.hrthm.2020.05.001 32387246 PMC7199677

